# A Cross-Sectional Study on the Cross-Talk of the COVID-19-Related Degree of Loneliness and the Etiological Factors Among the Elderly in Central China

**DOI:** 10.3389/fpsyt.2022.805664

**Published:** 2022-02-11

**Authors:** Jie Ju, Wen-bo Qi, Jia Zhang, Zhi-Jun Cao, Chi-Lun Tsai, Peng Liu

**Affiliations:** ^1^School of Physical Education and Training, Shanghai University of Sport, Shanghai, China; ^2^Department of Psychiatry, Qingdao Mental Health Center, Qingdao, China; ^3^Basic Courses Department, Shanghai I&C Foreign Languages School, Shanghai, China

**Keywords:** COVID-19, elderly, loneliness, sleep quality, quality of life, physical activities

## Abstract

**Background:**

The outbreak of COVID-19 has undoubtedly influenced the normal lifestyle of people worldwide, including the Chinese population. This study attempted to do supplementary research to the current situation of loneliness as well as the related risk factors among the elderly in the province in central Chinese during the COVID-19.

**Methods:**

We conducted a cross-sectional study in one of the central Chinese provinces (Henan Province) from December 2020 to March 2021 using a multistage sampling method, and 568 elderly people without cognitive impairment were interviewed. The UCLA Loneliness Scale, Pittsburgh Sleep Quality Index (PSQI), Physical Activity Rating Scale (PARS-3), and Quality of Life Questionnaire SF-36 were adopted to collect information. We used univariate and multivariate logistic regressions to analyze the factors resulting in severe loneliness among the elderly with seldom or regular participation in physical exercises.

**Results:**

During the epidemic in central China, the elderly suffering from loneliness syndrome accounted for 34.2%, of which 15.5% were severely lonely. Risk factors for severe loneliness were quality of life (OR: 7.129), sleep quality (OR: 3.778), seldom exercise (OR: 4.170), poor economic status (OR: 1.769), and negative attitude toward the prospects for the epidemic control (OR: 4.033). By grouping the participants in terms of physical activity, we found that the quality of life (OR:5.778) was a significant risk factor than sleep quality (OR:2.939) in the seldom exercise group, while the only risk factor in the regular exercise group was the quality of life (OR: 5.021).

**Conclusion:**

There was an increase in the degree of loneliness among the elderly during the epidemic, and physical activity played an active role in relieving the severe loneliness of the elderly. Therefore, for the sake of the elderly, regular participation in physical exercises should be encouraged during the duration of the epidemic.

## Background

Loneliness can be defined as one's emotional and psychological stress due to isolation from social activities or living in an unfrequented place (1). Loneliness is one of the important indicators for evaluating social health, and it is also a major public health problem (2). There are European countries that appoint ministers of loneliness specialized in dealing with the issue of social loneliness (3). The sense of loneliness mainly affects physical health and psychological behavioral mechanisms. This in turn exerts influence on physiological functions, neuroendocrine effects, perception of stressful events, immune function, sleep quality and healthy behaviors etc (1, 4). Heavier loneliness predict exaggerated responses to acute stress, leading to elevated interleukin-6 (IL-6), interleukin-1 beta (IL-1B), monocyte chemoattractant protein 1 (MCP-1), tumor necrosis factor alpha (TNFa), proinflammatory cytokines and glycoprotein etc. These biomarkers are associated with the incidence of cardiovascular disease (5–9). The sense of loneliness simultaneously reduces the quality of life (10), and increases the risk of illness and all-cause mortality (11–13). The psychological impact of loneliness is more obvious (14–16). Greater loneliness stimulates neuroendocrine dysregulation (17). By reducing the dendritic branches of the hippocampus and prefrontal cortex, it triggers the long-term activation of the hypothalamic–pituitary–adrenal axis (the HPA axis), thereby reducing nerve reserves, resulting in a decrease in memory and learning ability, and a decrease in cognitive function, leading to dementia and Alzheimer's disease (18). Other psychological problems caused by loneliness include depression, anxiety, schizophrenia, suicide, etc (4, 15, 19). Persistent loneliness is, however, associated with worse health outcomes, depression, psychiatric disorders, and behavioral abnormality among older adults (20). Studies have shown that longer periods of loneliness may worsen depression, anxiety, aggressive behavior, and cognitive complications (21). Other studies have also supported the fact that loneliness can increase the rate of morbidity and mortality by affecting both physical and mental health in critically ill patients, including individuals suffering from cardiovascular disorders (4, 22). Thus, the efforts to minimize the loneliness-related psychological stresses are not only important for research purposes but also crucial for improving the quality of life in the elderly, especially those who are terminally ill or suffer from irreversible degenerative diseases. Therefore, monitoring and investigating the exact reasons for loneliness is notable and meaningful in the context of geriatric healthcare support (20, 23).

With the outbreak of the COVID-19 pandemic, all the countries have been confronted with great challenges globally. World Health Organization (WHO) and Center for Disease Control and Prevention (CDC) believe that social distancing is one of the most efficient prevention strategies (24), despite of the result of social isolation. Different from loneliness, social isolation is an objective separation, while loneliness is a subjective separation (25). Both social isolation and loneliness have been shown to be harmful to health, such as reducing a healthy lifestyle, causing physiological reactions such as increased blood pressure and increased inflammatory response to stress (26). Studies have shown that social isolation has a greater impact on mortality than loneliness (29% vs. 26%) (27, 28). Conducted research from the perspective of brain neural mechanism, study reported individual subjectived perception of social isolation is one of the important risk factors of mortality in humans (17). Several studies have shown that social isolation will activate the hypothalamic-pituitary-adrenocortical axis, and it negatively affect a wide range of physiological, behavioral, and health outcomes (29, 30). During a 20-year follow-up investigation, the researchers found that the effects of loneliness and social isolation are synergistic: the impact of loneliness increases with the increase in the degree of social isolation, and the impact of social isolation on health also increases with the increase in the degree of loneliness (31, 32). This synergy has been confirmed in previous epidemiological retrospective studies. According to a study of 6, 231 South Korean residents who were quarantined for 2 weeks during the 2015 Middle East respiratory syndrome (MERS) pandemic (33) as well as another study of 1, 656 Koreans who were quarantined for 2 weeks (34), and an online survey of the quarantined people during the SARS pandemic, problems in varying degrees emerged about the physical and mental health of the quarantined (35) and increased with the lengthening of the quarantine time. It's worth noting that all interviewees described a sense of isolation. These observations are consistent with the results of studies in many countries during past disease (e.g., SARS, Ebola, H1N1 influenza, and Middle East respiratory syndrome) outbreaks. Social isolation causes the loss of normal contact with others, resulting in a sense of loneliness, as well as increased levels of stress, fear, and depression (36). Similarly, another retrospective study of the above-mentioned major epidemics shows that social isolation and loneliness have caused the occurrence of higher mortality rates to the elderly (25). Amongst all the age groups, the elderly population has been the most vulnerable and predisposed to high risk for COVID-19 infection and has accounted for the highest mortality rate, with or without any comorbid complications (37). In the United States, for example, 78% of the COVID-19-related deaths occurred in the age group of 65 years and over. In addition, the highest death toll among the elderly can be attributed to the age-associated declined immunity and/or suppressed immunity due to chronic comorbid complications, leading to the quick infection during the COVID-19 pandemic. Moreover, a significant portion of the elderly suffer from neurological and neuropsychiatric disorders and are most likely to remain in the persistently inflamed condition, resulting in the worst treatment outcomes once contracted COVID-19 (38). According to the global population census, China has the largest aged population in the world, where individuals aged 60 years and over occupy 18. Seventy percentage of the total population (39). In view of the present pandemic situation worldwide, controlling and preventing the pandemic outbreak will take a comparatively longer time in China. The prolonged pandemic may lead to most costly psychological cost (25). when the aged individuals are considered, social isolation and induced loneliness are more likely to cause negative impacts on their physical, social and psychological health, resulting in poor quality of life (38, 40, 41). Therefore, unlike the younger population, a slight rise in the duration of the lonely period during the pandemic may have dire consequences for the elderly (42).

Loneliness is one of the key challenges that must be addressed during the Covid-19 pandemic. As the epidemic continues, the degree of loneliness may increase (2). The elderly experienced social isolation and loneliness during the epidemic prevention and control period. The dual pressure of physical and psychological may cause more severe loneliness, and the health status of the elderly who have previously suffered from mental illness may be over-magnified (43). Even if when young people are under the dual effects of loneliness and social isolation, their antibody response to influenza immunity will turn worse, and the antibody response will become the lowest (44). In previous studies on factors related to loneliness in the elderly, sleep disorders, self-health evaluation, education level, socioeconomic status, physical activity, etc. are all associated with loneliness, while sleep disorders, self-health evaluation, physical activity have a two-way correlation with loneliness (15, 23, 45–48). Among the studies on interventions for loneliness, most studies include more social support, maintain social network, one-to-one interventions, supported living group interventions, community-based group interventions, new technologies (such as mobile communication, TV, Internet, etc.) (36, 49–51), shared activity plan (such as exercise, adult learning, etc.), traveling (52), playing games (43), doing sports, psychological therapies, social service, animal therapy, befriending and skill development etc (53). However, during the epidemic prevention and control period, limited social resources and social isolation requirements have restricted most interventions. A review study demonstrated that most of the physical activities of the elderly were accumulated through tourism, but this was unrealistic during the epidemic. In addition, although the use of mobile phones and the Internet can alleviate loneliness, it is reported that the elderly are less inclined to use technical communication to make social connections (54). Physical activity has a moderating effect on the elderly's sense of loneliness, but most of the researches mainly focus on the elderly's daily physical activities, not on purposeful physical exercise. A regression analysis study conducted by the University of London showed that loneliness was negatively correlated with daily physical activity. However, when covariates such as gender, age, education level, health status, and economy were added, loneliness had nothing to do with physical activity (52). It is necessary to conduct research on subjective physical exercise rather than daily physical activity of the elderly during the epidemic, because continuous physical activity can reduce cardiovascular risk, reduce disability and weakness, and enhance the independence and quality of life of the elderly (55). Thus, the present study aimed to investigate the current situation of loneliness among the elderly undergoing COVID-19 prevention and cure and assess the influence of the etiological factors like age, gender, education status, quality of life, sleep quality, income, exercise participation, and attitude toward the prospects for epidemic control, etc. Furthermore, we conducted the analysis to evaluate the impact of physical exercise on the management of loneliness in the elderly by grouping the study participants according to the etiological factors. In the light of this analysis, certain physical exercise therapies were suggested in order to effectively intervene in the loneliness syndrome in the elderly during the COVID-19.

## Materials and Methods

### Study Area and Participants

This study was conducted from December 2020 to March 2021. Henan Province was selected as the research area in this study for its geographic location in China. As a populous area located in central China, Henan Province had always been China's transportation hub and the most migrated area in central China. It definitely became one of the most populated provinces undergoing tremendous pressure for epidemic prevention and control since the outbreak of the COVID-19 pandemic. The surveyed areas are distributed in three sections of Henan: Pingdingshan, Xinyang, and Nanyang. Pingdingshan is located in the central part of Henan Province. Xinyang is 150 kilometers from Wuhan, Hubei Province, and Nanyang is 140 kilometers from Xiangyang, Hubei Province. Strict prevention and control measures were implemented in all of the three areas. Therefore, we believe that the selected research area is representative.

The inclusion criteria of the targeted population were: (1) age ≥ 60 years; (2) permanent resident; (3) not diagnosed with Alzheimer's disease (AD); (4) having clear awareness and can communicate with investigators without barriers, and (5) understood the contents of this survey and agreed to participate with voluntary cooperation. While the exclusion criteria included: (1) non-resident population; (2) elderly subjects suffering from any acute disease during the investigation; (3) elderly subjects with spinal or lower limb fractures in the past 6 months; (4) elderly subjects who couldn't walk independently; and (5) elderly persons suffering from malignant tumors, chronic renal insufficiency, etc. We further confirmed with the family members of the elderly participants to rule out the diagnosis of AD. All participants signed the written informed consent prior to their participation, voluntarily accepted the interview, and completed the questionnaire designed to investigate the participants' cognitive function, demographic characteristics, (include age, gender, education status, family income, and whether living alone or with family members/friends, etc). The calculation based on the sample size was supposed to be 5–10 times the influencing factors that were estimated 38 totally. We set the total number of valid questionnaires to no <380, and planned to collect no <200 questionnaires in each region. In the end, a total of 608 questionnaires were collected, and 568 of them were valid.

### Survey Scales

#### UCLA Loneliness Scale

The UCLA Loneliness Scale has retest reliability of 0.89. It consists of 20 items, each of which is evaluated with a 4-level score system. Nine of the 20 items are evaluated in the opposite way. The total score of this scale ranges from 20 to 80, with the score ranges 20–34 indicating low-level loneliness, 35–48 for medium-level loneliness, and 49–80 indicating high-level loneliness, respectively. The higher score corresponded to the higher degree of loneliness, and a score of ≥ 49 was considered severe loneliness in this study (56, 57).

#### Quality of Life Questionnaire SF-36

The SF-36 is internationally recognized as a universal assessment system for measuring the quality of life with high reliability and reproducibility (58). The SF-36 general scale was divided into 8 dimensions, involving Physiological Functioning (PF), Role-Physical (RP), Bodily Pain (BP), Vitality (VT), Social Functioning (SF), Role-Emotional (RE), Mental Health (MH), and General Health (GH). For example, item 3 in the dimension of Physiological Functioning is to evaluate the ability to “bend over, bend knees and squat”, a score of 3 indicating no obstacles, a score of 2 indicating few obstacles, a score of 1 meaning many obstacles. The sum of the scores of the 8 dimensions is the total quality of life score. The higher the score was, the better the quality of life was. A total score of >117 indicated the good quality of life in this study (58, 59). In this study, the Cronbach α coefficient of the scale is 0.874

#### Pittsburgh Sleep Quality Index (PSQI)

PSQI is a self-reporting questionnaire assessing the quality of sleep over a one-month interval. Seven components were included in the scale concerning subjective sleep quality, sleep latency, sleep duration, habitual sleep efficiency, sleep disorders, use of sleeping medication, and daytime dysfunction. The total score of PSQI ranged from 0 to 21, with >7 indicating poor sleep quality or sleep disturbances (60). The scale is widely used in the assessment of sleep quality of all kinds of people, and has good reliability and validity. The Cronbach α coefficient is 0.84, and the retest reliability is 0.86 (61).

#### Physical Activity Rating Scale (PARS-3)

PARS-3 categorizes the level of participation in physical activity from the three dimensions, such as physical exercise frequency, exercise time, and the intensity of exercise, including 1 item in each dimension, and each of these dimensions is evaluated with a 5-level scoring system. The score of physical activity participation is presented as physical exercise intensity multiplied by exercise time and exercise frequency. The highest score for physical activity participation is 100 points, and the lowest is 0. Between the two extremes, the score of ≤4 points refers to the infrequent exercise participation, and the score of ≥43 points corresponds to the considerable amount of exercise. In this study, the Cronbach alpha coefficient of this scale was 0.85 (62).

### Sociodemographic Variables

Demographics of the participants were collected by a self-designed questionnaire survey documenting the participants' age, gender, economic status, education status, and attitude toward the prospects for COVID-19 control and so on. The survey was conducted on a voluntary basis.

### Quality Control

Four investigators were recruited to conduct the survey, who received standardized training involving the principles and the strategies of conducting the survey prior to the study. The training involved making identical conversations, use of suggestive language, choice of time and location to distribute the questionnaire, and strategies of distributing the questionnaire. Moreover, considering the realistic fact that some of the elderly might not be able to read, we specified common descriptive language. During the entire process of the survey, we strictly abode by the local policies and regulations for COVID-19 prevention and control. The purpose of all the efforts was to ensure that the survey was performed and completed under the same experimental conditions.

### Statistical Analysis

In this study statistical analysis on the data was performed by using SPSS statistical data 25.0. Data entry into the computer was performed by two researchers repeatedly so as to ensure the accuracy of it. Sample data on demographic characteristics and related factors affecting loneliness of the elderly were described by frequency and percentage. The degree of loneliness in the elderly who participated in the survey was regarded as the dependent variable, and the content in the general data was regarded as the independent variable. In addition, the elderly were investigated by being grouped into regular exercise group and seldom/infrequent exercise group. Binary unconditional logistic regression was firstly conducted to make univariate analysis, and then multivariate regression analysis was performed on the factors with significant results by adjusting age as a fixed factor. The OR value of the factors affecting the loneliness of the elderly and their respective 95% confidence intervals (95% CI) and *P* values were calculated. *P* < 0.05 was considered statistically significant.

## Results

### Subject Characteristics

A total of 608 questionnaires were distributed during the study period, while 568 questionnaires were finally completed. There are 336 women, accounting for 59.1% of the total number. The age of the survey subjects ranged from 60 years old to 90 years old, and the proportion of elderly people between 60 and 75 years old occupies the highest 85%.102 among them had an education background of university or above, accounting for 18% of the total number. The number of people with poor economic status was 118, accounting for 20.8% of the total number. There were 342 people with a better quality of life, accounting for 60.2% of the total number 394 people had better sleep quality, accounting for 69.4% of the total. According to the result of the physical activity survey, the rates of regular exercise and seldom exercises were 80.1% and 19.9%, respectively. During the COVID-19 period, the proportion of loneliness among the elderly in the central province of China accounted for 34.2%, of which 15.5% were found severely lonely. According to the survey of attitude toward the prospects for the prevention and control of COVID-19, 12% of the participants showed a negative attitude (Table 1).

**Table 1:** Demographic details of the study participants.

**Factors**		***N* = 568**	**%**
Age group	>75	85	15
	60 to 75	483	85
Gender	Female	336	59.1
	Male	232	40.9
College or above	Yes	102	18
	No	466	82
Poor economic status^*^	Yes	118	20.8
	No	450	79.2
Good quality of life^*^	Yes	342	60.2
	No	226	39.8
Good sleep quality^*^	Yes	394	69.4
	No	174	30.6
Regular Exercise^*^	Yes	455	80.1
	No	113	19.9
Severe loneliness^*^	Yes	88	15.5
	No	480	84.5
Negative attitude toward epidemic control	Yes	68	12
	No	500	88

* *Poor economic status indicates household income ≤50% of local GDP per capita, with “no” indicating household income>50% GDP per capita in the local area instead; SF-36 score ≥117 indicates good quality of life, while the score <117 indicates poor quality of life; PSQI ≤ 7 indicates good sleep quality, while PSQI > 7 indicates poor sleep quality; PARS-3 score >4 indicates regular participation in exercise, PARS-3 score ≤4 indicates seldom participation in exercise; UCLA score ≥49 indicates severe loneliness, UCLA score <49 indicates less loneliness*.

### Univariate Analysis of Related Influencing Factors of Severe Loneliness Among the Elderly

By the univariate logistic analysis of the influencing factors related to the degree of severity of loneliness in the elderly, we found that economic status, the quality of life, sleep quality, physical activity, and attitude toward the prospects for epidemic control were statistically significant contributors to the severity of loneliness in the elderly (*P* < 0.05), while age, gender, and education status were not significantly correlated with the degree of loneliness in the elderly. Risk factors for severe loneliness were the quality of life [odds ratio (OR): 7.129, 95% confidence interval (CI)], poor sleep quality (OR: 3.778, 95% CI), seldom/infrequent exercise (OR: 4.170, 95% CI), poor economic status (OR: 1.769, 95% CI), and negative attitude toward the epidemic control (OR: 4.033, 95% CI).

By comparing the univariate logistic regression of the loneliness severity-related etiological factors between the regular and seldom/infrequent exercise groups, we showed that the quality of life and sleep quality both had significant impacts on the severity of loneliness in the elderly. The most interesting aspect in the regular exercise group was that negative attitude toward the epidemic control exerted a greater influence upon the elderly suffering due to severe loneliness, compared with that of the seldom/infrequent exercise group (Table 2).

**Table 2:** Univariate logistic regression analysis of related influencing factors of severe loneliness among the elderly.

**Factors**		**Total**			**Regular exercise**			**Seldom exercise**		
		***n***	**Severe loneliness%**	**OR (95%CI)**	***n***	**Severe loneliness%**	**OR (95%CI)**	***n***	**Severe loneliness%**	**OR (95%CI)**
Age	>75	85	20	1.758 (0.993 3.112)	58	13.8	1.356 (0.602 3.055)	26	38.5	1.602 (O.651 3.947)
	60-75	483	14.5		398	10.6		86	31.4	
Gender	Male female	232 336	14.7 16.1	1.115 (0.700 1.777)	196 260	10.2 11.5	1.148 (0.631 2.089)	36 76	39 31.5	
Education status	NO	466	14.8	1.317 (0.752 2.306)	369	10	1.576 (0.798 3.112)	97	33	1.354 (0.443 4.135
College or above	YES	102	18.6		87	14.9		15	40	
Good quality of life	NO YES	226 342	8.4 20.2	7.129^***^ (4.146 12.256)	158 298	22.8 4.7	5.986^***^ (3.116 11.49)	73 39	45 12.8	5.610^**^ (1.971 15.964
Poor sleep quality	YES NO	174 394	28.7 9.6	3.778^***^ (2.364 6.036)	114 342	19.3 8.2	2.682^**^ (1.465 4.910)	60 52	46.6 19	3.675^**^ (1.561 8.651
Regular	NO	112	33.6	4.170^***^ (2.557 6.801)						
Exercise	YES	456	11							
Poor ecoNomic Status Negative attitude	YES NO YES NO	118 450 68 500	22 13.8 35, 3 12.6	1.769^*^ (1.061 2.949) 4.033^***^ (2.305 7.055)	90 366 30 426	16.7 9.6 26.7 10	1.891 (0.983 3.640) 3.325^*^ (1.393 7.933)	28 84 38 74	39 32 44 28	1.366 (0.563 3.313 2.043 (0.904 4.616

* *P<0.01;*

** *P<0.005;*

*** *P<0.001; CI, confidence interval; OR, Odds ratio*.

### Multivariate Analysis of Related Influencing Factors of Severe Loneliness Among the Elderly

The multivariate logistic regression analysis of the factors for severe loneliness in the elderly by setting age as a fixed factor indicated that the significant influencing factors toward the severe loneliness in the elderly with seldom exercise were quality of life (OR: 5.778, 95% CI: 1.731–19.281) and sleep quality (OR: 2.939, 95% CI: 1.134–7.615) (*P* < 0.05), while as for the elderly with regular exercise, the only influencing factor of severe loneliness was the quality of life (OR: 5.021, 95% CI: 2.521–10.036) (*P* < 0.05; Table 3).

**Table 3:** Multivariate analysis of related influencing factors of severe loneliness among the elderly in terms of physical activity.

**Factor**	**OR**	**95%CI**	***P***
**Seldom exercise (*****n*** **= 112)**			
Age^*^	1.03	0.965 1.100	0.373
Good quality of life (NO vs. YES)	5.778	1.731 19.281	0.004
Poor sleep quality (YES vs. NO)	2.939	1.134 7.615	0.026
**Regular exercise (*****n*** **= 456)**			
Age^*^	1.001	0.955 1.049	0.968
Good quality of life (NO vs. YES)	5.021	2.521 10.036	0.000

*Fixed in the model*.

* *Age was set as a continuous variable in the multivariate analysis*.

## Discussion

This study was mainly designed to study the level of loneliness among the elderly in the central Chinese province during the COVID-19 prevention and control period, concluding that the severe loneliness of the elderly was mostly related to the quality of life, sleep quality, economic status, and the physical status activity. The results of this study were consistent with previous research reports (45, 63, 64). Based on our findings, age, gender, and education status didn't have a significant correlation with the severity of loneliness among the elderly, despite the results of few studies showing that loneliness was highly correlated with age. This inconsistency might be attributed to the wider age ranges of the participants (20 vs. 50 vs. 80 years old) in those studies (21). To better understand the impact of age-associated etiological factors on the degree of loneliness, we selected the participants aged 60 years and above. In the light of our study, better economic status had a protective effect on the occurrence of loneliness, which was in line with the conclusion of the European research studies. However, earlier studies have revealed that compared with the elderly, loneliness exerted a higher impact on middle-aged people (50–59 years old) in poverty (65). This study also investigated the attitude of the elderly toward the prospects for COVID-19 control and found that only 12% of the elderly showed a negative attitude. Based upon the multivariate analysis, it was found that the attitude toward the prospects for the epidemic control did not increase the risk of severe loneliness, regardless of the group with regular exercise or seldom exercise.

It has been observed that the health and psychological complications due to loneliness are more serious than those before the epidemic (34.2 vs. 28%) in the background of COVID-19 among the elderly in the central China province (66). Studies from the United States and Europe have also indicated that loneliness-associated elderly suffering has been drastically growing during the period of acute outbreaks of COVID-19 (20, 42). According to the research performed by Groarke's team from the United Kingdom, poor sleep quality and difficulty in performing regular exercise were believed to be the major factors for loneliness during the initial lockdown period of the COVID-19 outbreak (67). The results of our research eventually backed up the hypothesis that sleep quality could have an impact on the occurrence of severe loneliness, which was similar to the results of the study conducted in Shandong Province in China (68). The impact of sleep on loneliness is mainly in terms of quality of sleep and sleep satisfaction and is irrelevant to the length of sleep (69).

According to other studies, critical reasons for the growing proportion of loneliness among the elderly during the COVID-19 pandemic have been the restricted social isolation and activities during the period of disease prevention and control measures. Social isolation was discovered to be primarily related to the degree of loneliness (42, 43), since separation from the outside world reduced the time the elderly spent on physical activities and thus brought about an increase in the sedentary time, eventually resulting in the decreased physical health and sense of happiness, and consequently a sharp decline in the quality of life (52). Physical activity has been found to play an active role in carrying out effective interventions in the loneliness of the elderly (64). It's also a practical means of improving old people's quality of life, physical health, and sleep quality (52, 70). Similarly, our research showed better sleep quality in the regular exercise group than in the seldom exercise group (53.6 vs. 25%). Furthermore, the quality of life for the elderly in the regular exercise group was also better than that in the seldom exercise group (65.2 vs. 34.6%). The multivariate regression analysis indicated that the main risk factors for the onset of severe loneliness in the elderly in the seldom exercise group were the quality of life and sleep quality, while the major risk factor for severe loneliness of the elderly in the regular exercise group was simply the quality of life. In other words, our results suggest that regular physical exercises function effectively to alleviate the severe loneliness in the elderly during and post-COVID-19 when the epidemic prevention and control measures are still in effect.

Although the advisable exercise time is considered to be 150 mins per week (64) and group exercises are assumed to have a better impact on the intervention in the loneliness of the elderly (71), regular participation in physical activities and the implementation of group exercises during COVID-19 prevention and control were difficult tasks than during non-epidemic period. In view of this situation and based upon the results of our study, it is suggested that the elderly should maintain a minimum amount of exercise as 1–2 times a week for 21–30 mins of walking or calisthenics with radio music, or 1–2 times a week for 11–20 mins of jogging and so on, for the purpose of relieving or reducing the stress of loneliness. In addition, social interactions through the internet and group fitness exercises with the aid of communication tools have also been proved helpful in reducing loneliness among the elderly (72).

There inevitably existed some limitations in this study. Firstly, it was restricted to the cross-sectional design. Secondly, since Henan province was the only selected study area, the survey data from other provinces were lacking. Therefore, follow-up studies involving more provinces in the future are supposed to be conducted to further validate our findings.

This was a pilot study to evaluate and assess the current situation of loneliness among the elderly in central China and the related influencing factors during COVID-19. The COVID-19 pandemic definitely increased the severity of loneliness among the elderly. It takes a long, long time and more effort to improve the quality of life of the elderly. The intervention of physical activities is often easier and may be effective in a short time. On the premise of adhering to the epidemic prevention and control measures, physical activity intervention was a more practical and effective short-term means compared with improving the quality of life and sleep quality of the elderly. In summary, encouraging the elderly to participate in physical activities may have positive effects on their quality of life and also play an active role in reducing the risk of severe loneliness.

## Data Availability Statement

The original contributions presented in the study are included in the article/supplementary material, further inquiries can be directed to the corresponding author/s.

## Ethics Statement

Ethical review and approval was not required for the study on human participants in accordance with the local legislation and institutional requirements. The patients/participants provided their written informed consent to participate in this study.

## Author Contributions

PL, C-LT, and JJ designed the research protocol and performed the study. PL analyzed the data. W-bQ, JZ, and Z-JC performed the investigation. JJ drafted the manuscript. PL, JJ, C-LT, W-bQ, JZ, and Z-JC read and revised the manuscript. All authors read and approved the final version of the manuscript.

## Conflict of Interest

The authors declare that the research was conducted in the absence of any commercial or financial relationships that could be construed as a potential conflict of interest.

## Publisher's Note

All claims expressed in this article are solely those of the authors and do not necessarily represent those of their affiliated organizations, or those of the publisher, the editors and the reviewers. Any product that may be evaluated in this article, or claim that may be made by its manufacturer, is not guaranteed or endorsed by the publisher.
